# Esculetin Improves LPS/D-GalN Induced Acute Liver Injury Through AMPK/SIRT1/PGC-1α Signaling Pathway

**DOI:** 10.3390/antiox15050652

**Published:** 2026-05-21

**Authors:** Haoyang Dai, Qinqin Zhang, Pei Chen, Suiqing Chen

**Affiliations:** 1 College of Pharmacy, Henan University of Chinese Medicine, 156 Jinshui East Road, Zhengzhou 450046, China; 2 Henan Key Laboratory of Chinese Medicine Resources and Chemistry, 156 Jinshui East Road, Zhengzhou 450046, China; 3 Collaborative Innovation Center of Research and Development on the Whole Industry Chain of Yu-Yao, Zhengzhou 450046, China

**Keywords:** esculetin, acute liver injury, AMPK/SIRT1/PGC-1α, oxidative stress, mitochondrial function, apoptosis

## Abstract

The pathogenesis of acute liver injury (ALI) involves the loss of hepatic detoxification function, massive death of liver parenchymal cells within a short period, and excessive inflammatory responses. Studies have shown that Esculetin (Esc) possesses potent anti-inflammatory, antioxidant, and anti-tumor properties. In this study, we investigate whether Esc has a protective effect against ALI in mice and its potential mechanism. Esculetin markedly decreased ROS, MitoSOX, and apoptosis levels in AML12 cells and restored MMP. H&E staining demonstrated that Esc alleviated hepatic histopathological injury, and its intervention reduced serum ALT and AST levels. Moreover, Esc diminished ROS and apoptosis levels in the liver. Hepatic proteomic profiling identified the AMPK signaling pathway. Esc reduced the protein levels of p-AMPK/AMPK, PGC-1α, p-SIRT1/SIRT1, and BAX and upregulated the levels of Bcl-2 in liver tissue. Concomitantly, we added inhibitor Compound C (CC) to the AML12 cells to assess whether Esc acted through the AMPK pathway. The results showed that CC exacerbated the degree of liver injury, whereas Esc was able to reverse these phenomena, thus exerting an anti-liver injury effect. These findings provide mechanistic insights into the protective effects of Esc against ALI and support its potential as a therapeutic candidate for ALI.

## 1. Introduction

The liver plays a central role in metabolism, detoxification, and energy homeostasis [1]. With changes in lifestyles and improved living standards, the incidence of ALI has been rising annually, becoming the second leading cause of liver injury after viral hepatitis. Acute liver injury is caused by various factors, such as chemical agents, alcohol consumption, viral infections, and metabolic disorders. It is characterized by the rapid loss of functional hepatocytes [2,3]. Uncontrolled ALI can lead to irreversible liver damage and compensatory repair, resulting in liver fibrosis and ultimately progressing to cirrhosis or hepatocellular carcinoma. Currently, liver diseases account for approximately 2 million deaths worldwide each year [4]. Due to the incomplete understanding of the underlying mechanisms, liver transplantation remains the only definitive treatment, in addition to supportive therapies such as intensive care and nutritional intervention [5]. However, its clinical application is limited by donor shortages and poor post-transplant outcomes [6]. Therefore, elucidating the pathogenesis of ALI and developing effective hepatoprotective agents are of great clinical significance.

AMPK is a key regulator of cellular energy metabolism and is widely expressed in metabolically active tissues, including the liver, skeletal muscle, and adipose tissue [7,8,9]. By sensing changes in the intracellular AMP/ATP ratio, AMPK coordinates multiple physiological and pathological processes, such as energy metabolism, oxidative stress, inflammatory responses, and apoptosis, and is a key molecule in maintaining cellular homeostasis [10]. As the central organ of systemic metabolism, the liver relies heavily on energy balance to sustain its physiological functions [11]. Notably, the AMPK signaling pathway is closely associated with oxidative stress regulation and mitochondrial function and plays a pivotal role in hepatic lipid metabolism, mitochondrial biogenesis, and the progression of liver injury-related diseases [12,13]. Recent studies have demonstrated that AMPK activation exerts significant therapeutic effects on non-alcoholic fatty liver disease (NAFLD) [14]. As a natural coumarin compound, Esculetin (Esc) possesses potent anti-inflammatory, antioxidant, and anti-tumor properties [15,16], which are theoretically relevant to the pathogenesis of liver injury. However, whether Esc exerts its protective effects through modulation of the AMPK signaling axis has not been systematically investigated in ALI. The LPS/D-GalN-induced animal liver injury model has a high pathophysiological similarity to human acute liver injury [17]. Therefore, this study employed this model to evaluate the therapeutic effects of Esc on liver injury. Furthermore, we investigated whether these effects are mediated through the AMPK/SIRT1/PGC-1α signaling pathway, aiming to clarify the molecular mechanisms underlying the protective effects of Esc in ALI.

## 2. Materials and Methods

### 2.1. Materials and Reagents

The JC-1 Kit (G1515-100T), Fluorescein (FITC) TUNEL Cell Apoptosis Detection Kit (G1501), and MitoSOX Red Kit (G1734-20UL) were purchased from Servicebio (Wuhan, China). The ROS Kit was purchased from Solarbio (CA1410, Beijing, China). Assay kits for ALT and AST (C009-2-1, C010-2-1, E003-2-1) were supplied by Nanjing Jiancheng (Nanjing, China). The positive drug (Y, Shuifei Jibin Jiaonang) was acquired from Tianjin Tisly Sants Pharmaceutical Co., Ltd. (Tianjin, China). The D-GalN (G115553) was purchased from Shanghai Aladdin Biochemical Technology Co., Ltd., Shanghai, China. The LPS (297-473-0) was purchased from Merck (Darmstadt, Germany). Esculetin (305-01-1) was purchased from MCE (Monmouth Junction, NJ, USA).

### 2.2. Cell Culture

Mouse normal liver cells (AML12) were obtained from Wuhan Procell (Wuhan, China). These cells were cultured in DMEM/F12 supplemented with 10% FBS (fetal bovine serum), 1% penicillin–streptomycin (P/S), 0.5% ITS-G, and 40 ng/mL Dexamethasone, and maintained at 37 °C in a 5% CO_2_ incubator. LPS-stimulated AML12 cells were treated with Esc for 24 h, and then the cells were collected for subsequent assays.

The ROS and MitoROS levels of AML12 were assessed with DCFH-DA and MitoSOX Red kits, respectively. The level of MMP was measured by the JC-1 kit [18]. The level of apoptosis was measured by TUNEL staining. In accordance with the manufacturer’s instructions for the corresponding reagent kits, images were captured and analyzed using a fluorescence microscope.

### 2.3. Animals

6–8 week-old male BALB/c mice were purchased from Zhejiang Vital River Laboratory Animal Technology Co., Ltd. (Pinghu City, China). All mice were housed in captivity under standard animal housing conditions with ad libitum access to food and water. The animal experiment was approved by the Experimental Animal Ethics Committee of Henan University of Chinese Medicine (Ethical Review Approval No. IACUC-202402006, 19 February 2024–19 February 2029). The experimental process strictly abided by the relevant provisions of the ARRIVE guidelines.

Fifty mice were randomly divided into five groups as follows: the control group (CON, *n* = 10), the model group (M, 700 mg/kg D-GalN and 10 μg/kg LPS, *n* = 10), the model + positive drug group (Y, Shuifei Jibin Jiaonang 48 mg/kg, *n* = 10), the model + low-dose esculetin group (20 mg/kg, Esc-L, *n* = 10), and the model + high-dose esculetin group (40 mg/kg, Esc-H, *n* = 10). Group Y, the Esc-L group, and the Esc-H group received corresponding drug treatments by gavage for 7 days. On day seven, one hour after gavage, all mice received an intraperitoneal injection of 700 mg/kg D-GalN and 10 μg/kg LPS, and only mice in the CON group received saline. After 6 h, blood and liver were taken for subsequent testing.

### 2.4. HE Staining

Liver tissues of each group of mice were fixed in 4% paraformaldehyde for 24 h. After gradient dehydration with ethanol, the tissues were embedded in paraffin and sectioned for hematoxylin and eosin (HE) staining. The sections were observed under a microscope.

### 2.5. Biochemical Assays in Serum

Mouse blood was collected and centrifuged at 3000 rpm for 15 min at 4 °C. The levels of AST, ALT, and TBA were measured according to the kit instructions.

### 2.6. ROS Detection

Following dewaxing, paraffin sections were incubated with a ROS fluorescent probe at 37 °C for 30 min. After three 5 min washes with PBS, sections were stained with DAPI at room temperature for 10 min, washed another three times with PBS (5 min each), and mounted with an antifade mounting medium.

### 2.7. TUNEL Staining

Slides were dewaxed in dewaxing solution I and II for 30 min each, then dehydrated in anhydrous ethanol I and II, 95%, 85%, and 75% ethanol for 5 min each, followed by rinsing with tap water for 5 min. Antigen retrieval was performed in citrate buffer using a microwave. A fluorescent TUNEL reaction mixture (A:B = 1:30) was prepared in the dark, and slides were incubated at 37 °C for 1 h. After PBS washing, nuclei were counterstained with DAPI for 15 min, rinsed with PBS, and coverslipped.

### 2.8. DIA Proteomics

Liver tissue samples were removed under frozen conditions and transferred to MP shaking tubes, and protein lysis buffer was added. The samples were shaken and lysed on ice for 30 min. After centrifugation at 4 °C and 12,000× *g* for 30 min, the supernatant was collected. Protein content was determined using the BCA method, followed by SDS-PAGE electrophoresis. A suitable amount of protein sample was subjected to TCEP reduction, iodoacetamide alkylation, and acetone precipitation. The sample was then digested overnight with trypsin. The digested products were desalted, quantified, and detected using a Vanquish Neo chromatograph combined with an Orbitrap Astral mass spectrometer in DIA mode. Raw data were analyzed using Spectronaut™ 18 software. Bioinformatics analysis was performed using the “MajorBio Cloud Platform” (http://cloud.majorbio.com, accessed on 21 October 2025), and subsequent GO and KEGG analyses were performed.

### 2.9. Western Blot

Total protein was extracted with RIPA lysis buffer containing protease and phosphatase inhibitors. Protein concentration was measured by the BCA assay. Equal amounts of protein were separated by SDS-PAGE and transferred to PVDF membranes. Membranes were blocked with 5% BSA in PBST for 1 h at room temperature, then incubated with primary antibodies at 4 °C overnight, followed by HRP-conjugated secondary antibodies for 1 h at room temperature. Protein bands were detected by an ECL system, and band intensities were quantified using ImageJ software 1.54g.

### 2.10. Molecular Docking

The proteins were the AMPK protein (PDB: 2H6D), PGC-1α protein (PDB: 3FS1), and SIRT1 protein (PDB: 4IG9). These target proteins were imported into SailVina Final 1.0 to generate the corresponding .pdbqt files. The ligand file in .sdf format was also imported into SailVina Final 1.0 to generate its .pdbqt file. Molecular docking between the target proteins and the ligand was performed using SailVina Final 1.0 [19]. The docking results were further processed, analyzed, and visualized using OpenBabel-2.4.1, PyMOL-2.3.4, and Discovery Studio Visualizer v4.5.0.15071.

### 2.11. Immunofluorescence

Paraffin sections were dewaxed, rehydrated, and subjected to antigen retrieval. After blocking with BSA, the sections were incubated with diluted primary antibody against AMPK overnight at 4 °C. Following rinsing, the sections were incubated with the corresponding secondary antibody. The sections were then stained with DAPI at room temperature, mounted, and observed and photographed under a fluorescence microscope.

### 2.12. Immunocytochemistry of AML12 Cell

AML12 cells were allocated into four groups: CON, M (1.0 μg/mL LPS), Esc group (Esc, 10 μM Esc + 1.0 μg/mL LPS), and an AMPK inhibitor (compound C, CC) + Esc group (Esc + CC, 10 μM Esc + 5 μM CC + 1.0 μg/mL LPS) was established in parallel.

After discarding the culture medium, cells were fixed with formaldehyde and permeabilized with 0.25% Triton X-100. Subsequently, the cells were blocked with 1% BSA and incubated overnight at 4 °C with primary antibodies against p-AMPK, p-SIRT1, and PGC-1α. Following washing with PBS, the corresponding secondary antibodies were added and incubated. After rinsing with PBST, cells were stained with DAPI and coverslipped.

### 2.13. Statistical Analysis

Statistical analysis was performed using IBM SPSS 26.0 software (Armonk, NY, USA). One-way analysis of variance (ANOVA) was used to assess differences among groups. Post hoc multiple comparisons were performed using the least significant difference (LSD) test. A probability level of *p* < 0.05 was defined as statistically significant, and *p* < 0.01 as highly statistically significant.

## 3. Results

### 3.1. Study on the Interventional Effects of Esc on AML12 Cells

**Figure 1:**
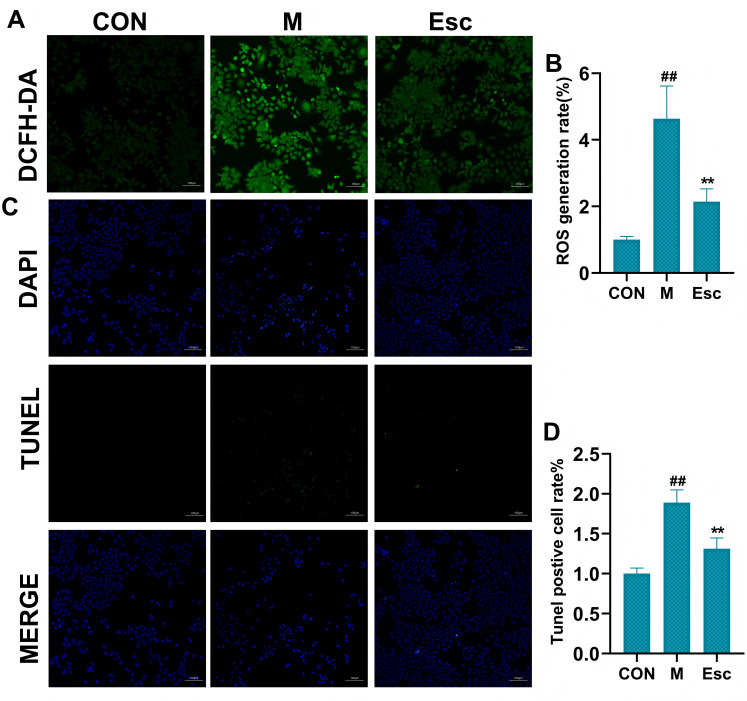
Effects of Esc on LPS-induced AML12 cells. CON: the control group; M: the model group; Esc: the model group + the Esc group. (**A**) Levels of ROS. (**B**) ROS generation rate. (**C**) Levels of TUNEL. (**D**) TUNEL positive cell rate. Data are presented as mean ± SD. ^##^ *p* < 0.01 compared with the CON group; ** *p* < 0.01 compared with the M group.

**Figure 2:**
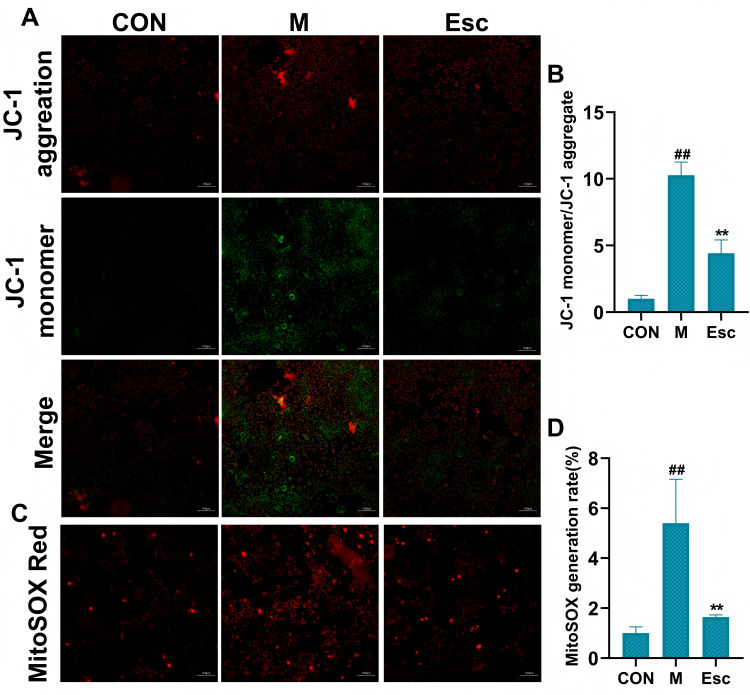
Effects of Esc on LPS-induced AML12 cells. CON: the control group; M: the model group; Esc: the model group + the Esc group. (**A**) Levels of JC-1. (**B**) JC-1 monomer/JC-1 aggregate. (**C**) Levels of MitoSOX Red. (**D**) MitoSOX generation rate. Data are presented as mean ± SD. ^##^ *p* < 0.01 compared with the CON group; ** *p* < 0.01 compared with the M group.

As shown in Figure 1A,B, the fluorescence intensity was markedly enhanced in the M group, whereas Esc intervention significantly attenuated the fluorescence signal, indicating a remarkable reduction in intracellular ROS accumulation. Regarding cell apoptosis, TUNEL-positive cells were significantly increased in the M group, suggesting severe apoptosis induced by LPS in AML12 cells. In contrast, the number of TUNEL-positive cells was markedly decreased in the Esc-treated group, demonstrating that Esc effectively alleviated LPS-induced apoptotic injury in AML12 cells (Figure 1C,D). Furthermore, mitochondrial function was evaluated. The M group exhibited a distinct shift from red to green fluorescence, indicative of mitochondrial membrane potential (MMP) depolarization and dysfunction. Notably, Esc treatment effectively reversed the LPS-induced loss of MMP and preserved mitochondrial function (Figure 2A,B). Additionally, the M group exhibited a pronounced increase in red fluorescence intensity, indicative of excessive mitochondrial superoxide production. Esc treatment significantly reversed the LPS-induced elevation of superoxide (Figure 2C,D).

### 3.2. Esc Attenuates LPS-Induced Liver Injury in Mice

**Figure 3:**
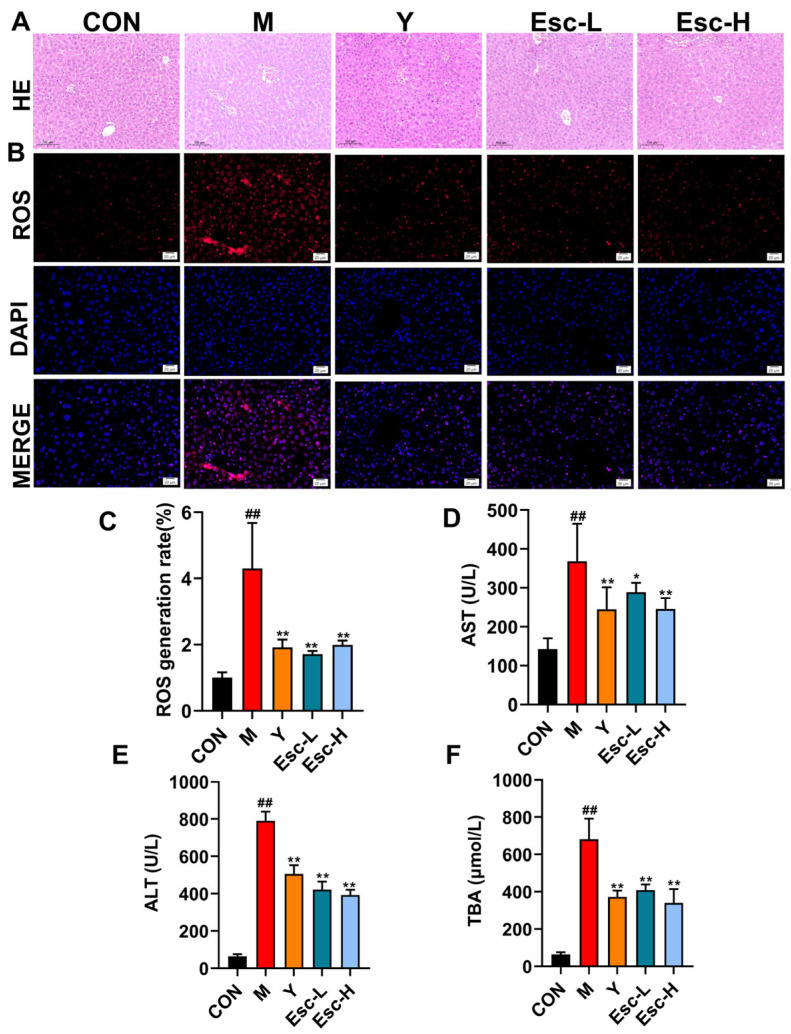
Protective effect of Esc on the liver in mice with ALI. CON: the control group; M: the model group; Y: the model + positive drug group; Esc-L: the model + low-dose esculetin group; Esc-H: the model + high-dose esculetin group. (**A**) HE staining of liver tissues. (**B**) ROS staining of liver tissues. (**C**) The level of ROS in the liver. (**D**–**F**) The levels of AST, ALT, and TBA in the serum. Data are presented as mean ± SD. ^##^ *p* < 0.01 compared with the CON group; * *p* < 0.05, ** *p* < 0.01 compared with the M group. Hematoxylin-eosin (H&E) staining. Scale bar, 100 μm; Immunofluorescence micrographs. Scale bar, 20 μm.

**Figure 4:**
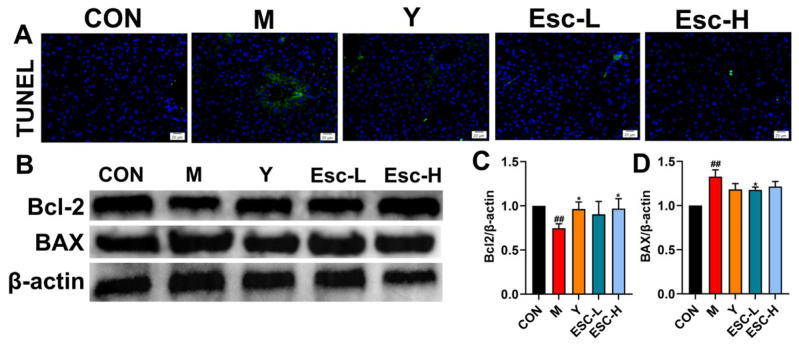
CON: the control group; M: the model group; Y: the model + positive drug group; Esc-L: the model + low-dose esculetin group; Esc-H: the model + high-dose esculetin group. (**A**) TUNEL staining of liver tissues. (**B**–**D**) Effects of Esc on the protein levels of Bcl-2/β-actin, BAX/β-actin. Data are presented as mean ± SD. ^##^ *p* < 0.01 compared with the CON group; * *p* < 0.05 compared with the M group. Immunofluorescence micrographs. Scale bar, 20 μm.

Histological analysis via H&E staining demonstrated that the CON group maintained intact hepatic architecture with distinct cell boundaries and no signs of inflammation. Conversely, the M group exhibited severe pathological alterations, characterized by extensive hepatocyte necrosis, massive inflammatory cell infiltration, and the disruption of hepatic cord structure. Notably, Esc treatment significantly alleviated these histopathological damages, preserving tissue integrity and reducing necrosis in a dose-dependent manner (Figure 3A). Consistent with these histological improvements, the M group showed significant increases in serum ALT, AST, and TBA levels compared with the CON group, while Esc administration markedly reduced these liver injury-related biochemical indicators (Figure 3D,F). Furthermore, hepatic ROS accumulation and hepatocyte apoptosis were sharply elevated in the M group, as verified by ROS and TUNEL staining; conversely, Esc intervention effectively reversed these abnormal changes (Figure 3B,C). Western blot analysis revealed a trend wherein Esc downregulated Bax and upregulated Bcl-2 compared to the M group (Figure 4B–D). Collectively, these findings demonstrated that Esc exerted hepatoprotective effects by inhibiting oxidative stress and hepatocyte apoptosis.

### 3.3. The Impact of Esc on Proteomics in Liver Tissue

**Figure 5:**
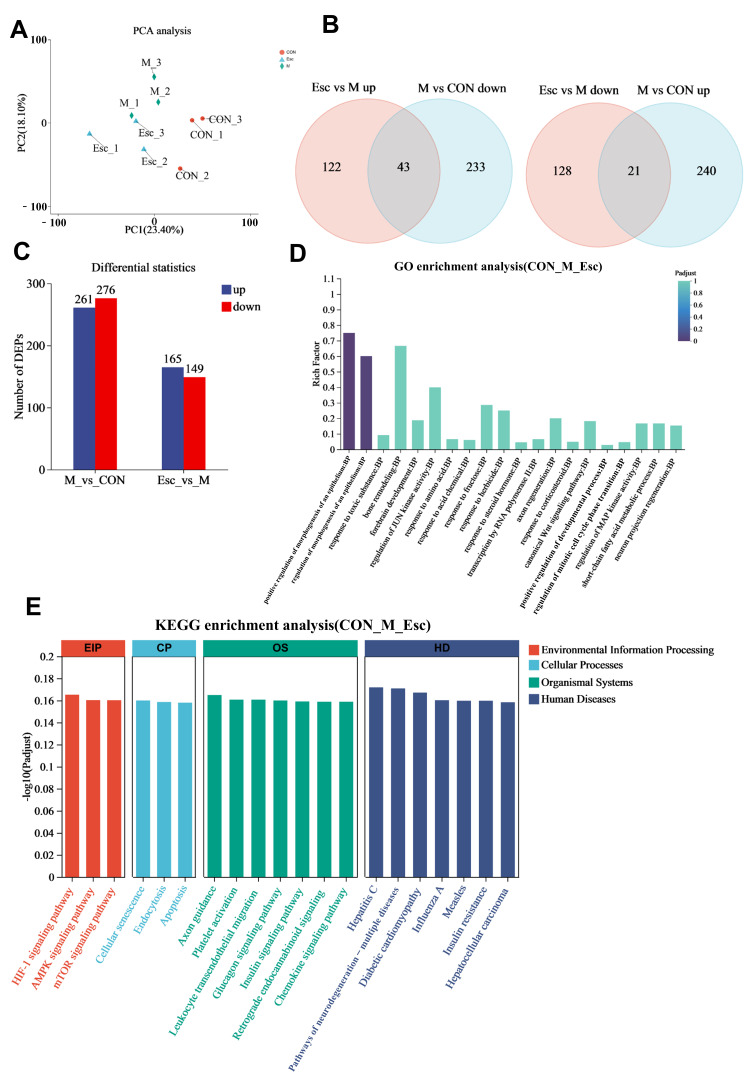
Effect of Esc on ALI in proteomic sequencing. CON: the control group; M: the model group; Esc: the model group + the Esc group. (**A**) PCA analysis. (**B**) Venn diagram. (**C**) Number of DEGs. (**D**) GO enrichment analysis. (**E**) KEGG enrichment analysis.

Protein expressions in the livers of the CON, M, and Esc groups were analyzed through proteomics. PCA results demonstrated that the CON, M, and Esc groups were clearly clustered into three distinct sets, indicating high intra-group repeatability and significant inter-group differences (Figure 5A). Compared with the CON group, 276 proteins were downregulated, and 261 proteins were upregulated in the M group; compared with the M group, the Esc group had 149 proteins downregulated and 165 proteins upregulated. Among these proteins, 64 proteins showed significant changes in the M group and were regulated by ESC (Figure 5B,C). GO enrichment analysis revealed that the differential proteins were mainly enriched in terms including positive regulation of morphogenesis of an epithelium, regulation of morphogenesis of an epithelium, bone remodeling, and others (Figure 5D). KEGG enrichment analysis showed that the AMPK signaling pathway, mTOR signaling pathway, apoptosis, and other pathways were significantly enriched (Figure 5E).

### 3.4. Molecular Docking Analysis

**Figure 6:**
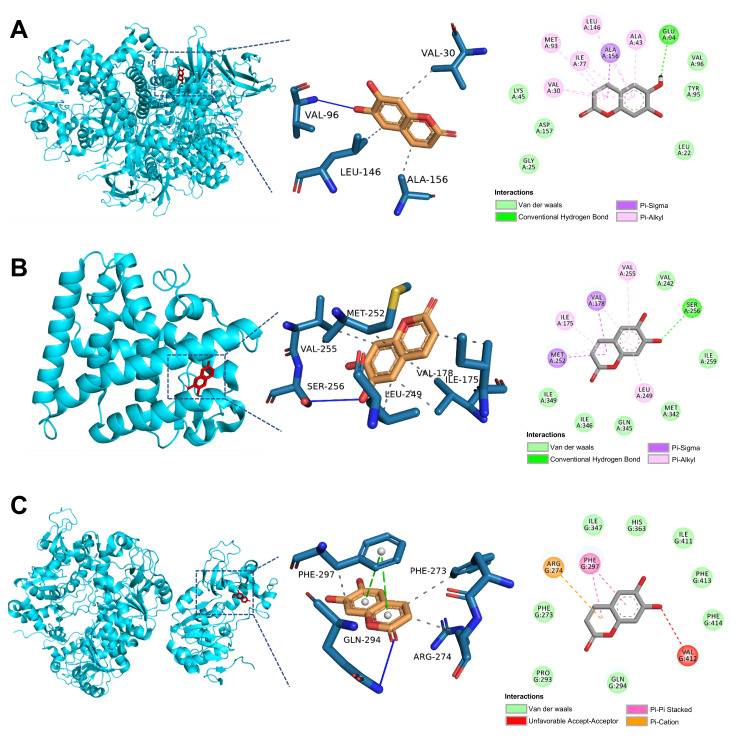
The docking score of Esc. (**A**–**C**) Molecular docking of Esc with AMPK, PGC-1α, and SIRT1 (2D and 3D).

A binding energy < −5.0 kcal/mol suggests theoretical potential binding rather than direct physical interaction. The molecular docking results indicated that the binding energies of Esc with AMPK, PGC-1α, and SIRT1 were −6.5 kcal/mol, −6.4 kcal/mol, and −6.2 kcal/mol, respectively (Figure 6A–C, Table 1).

### 3.5. Esc Improves Liver Injury Through AMPK/SIRT/PGC-1α Signaling Pathway

**Figure 7:**
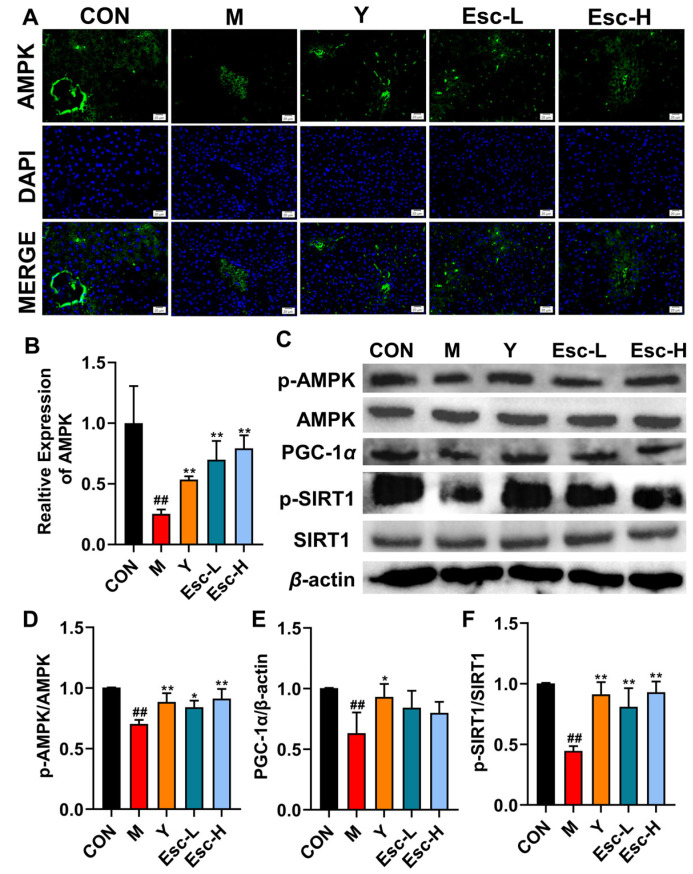
Effects of Esc on AMPK/SIRT1/PGC-1α. CON: the control group; M: the model group; Y: the model + positive drug group; Esc-L: the model + low-dose esculetin group; Esc-H: the model + high-dose esculetin group. (**A**) Immunofluorescence staining for AMPK. (**B**) The levels of AMPK. (**C**–**F**) Effects of Esc on the protein levels of p-AMPK/AMPK, PGC-1α, p-SIRT1/SIRT1. Data are presented as mean ± SD. ^##^ *p* < 0.01 compared with the CON group; * *p* < 0.05, ** *p* < 0.01 compared with the M group. Immunofluorescence micrographs. Scale bar, 20 μm.

The expression of AMPK in liver tissues was detected by immunofluorescence staining. As shown in Figure 7A,B, compared with the CON group, the green fluorescence intensity of AMPK was significantly decreased in the M group, whereas Esc treatment markedly increased AMPK expression. Western blot analysis was performed to examine the protein levels of p-AMPK, AMPK, PGC-1α, p-SIRT1, and SIRT1. Compared with the CON group, the phosphorylation and expression levels of these key proteins were significantly downregulated in the M group, and this reduction was effectively reversed by Esc intervention (Figure 7C–F).

### 3.6. Esc Improves Liver Injury Through the AMPK Signaling Pathway

**Figure 8:**
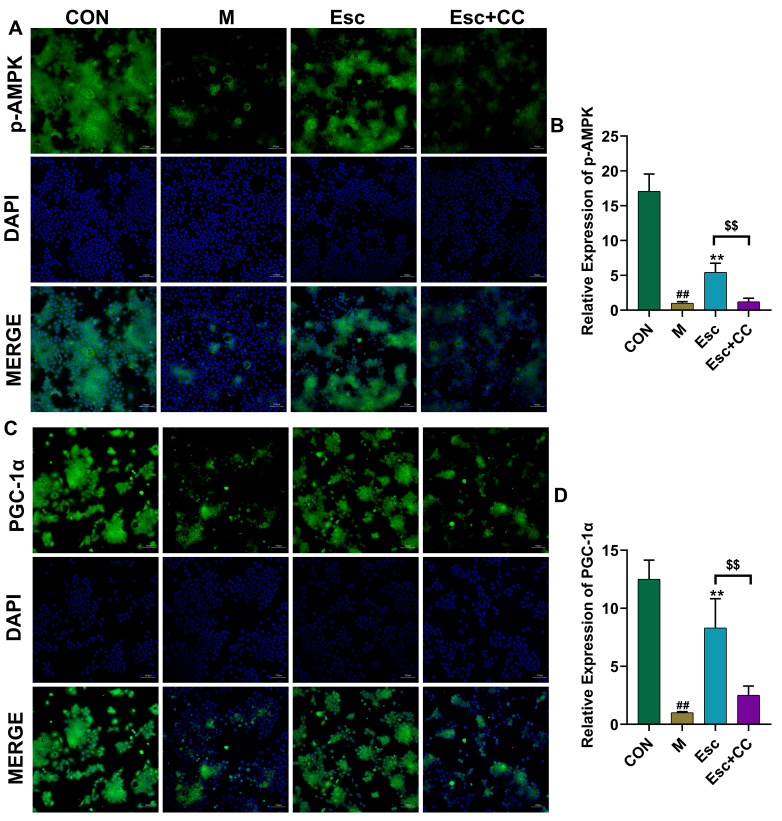
CC weakened the protective effect of Esc against AML12 cells. CON: the control group; M: the model group; Esc: the model group + the Esc group; Esc + CC: the model group + the Esc group + Compound C. (**A**,**C**) Immunocytochemistry staining for p-AMPK and PGC-1α. (**B**,**D**) The levels of p-AMPK and PGC-1α. Data are presented as mean ± SD. ^##^ *p* < 0.01 compared with the CON group; ** *p* < 0.01 compared with the M group; ^$$^ *p* < 0.01 compared with the ESC group.

**Figure 9:**
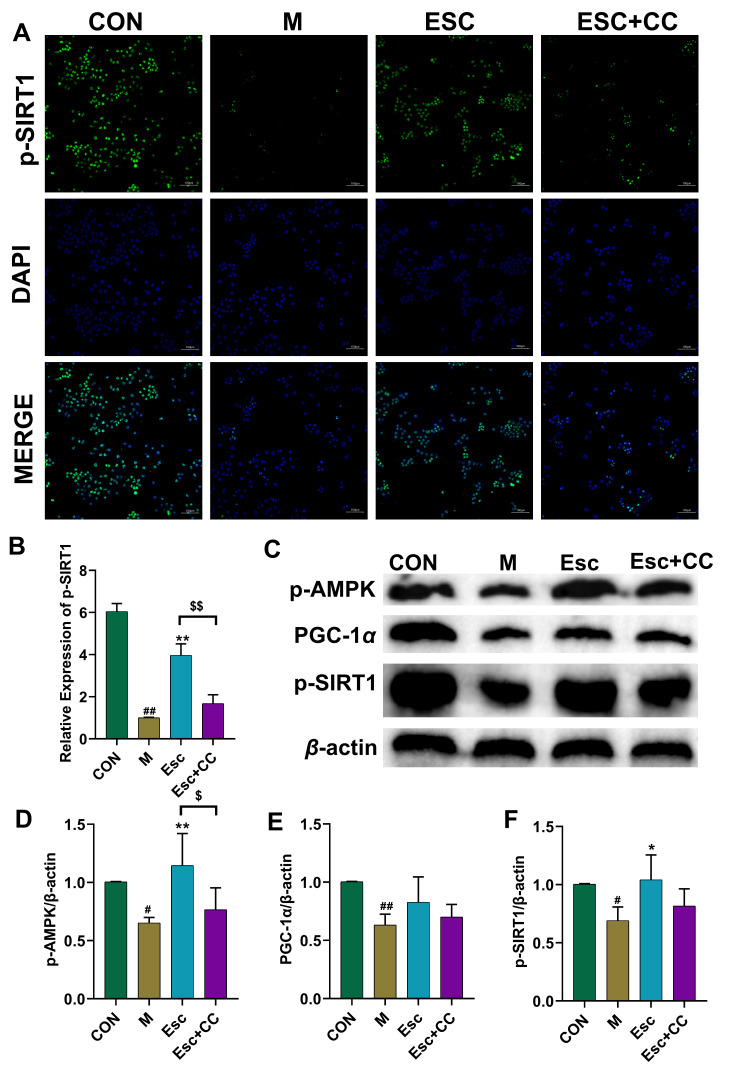
The relationship between the effects of Esc on AML12 cells and the AMPK signaling pathway. CON: the control group; M: the model group; Esc: the model group + the Esc group; Esc + CC: the model group + the Esc group + Compound C. (**A**) Immunocytochemistry staining for p-SIRT1. (**B**) The levels of p-SIRT1. (**C**–**F**) Effects of Esc on the protein levels of p-AMPK, p-SIRT1, and PGC-1α. Data are presented as mean ± SD. ^#^ *p* < 0.05, ^##^ *p* < 0.01 compared with the CON group; * *p* < 0.05, ** *p* < 0.01 compared with the M group; ^$^ *p* < 0.05, ^$$^ *p* < 0.01 compared with the ESC group.

To investigate the potential involvement of the AMPK signaling pathway in the hepatoprotective effect of Esc against liver injury, the AMPK inhibitor Compound C (CC) was applied to AML12 cells. As shown in Figure 8A–D and Figure 9, compared with the CON group, the expression levels of p-AMPK, p-SIRT1, and PGC-1α were significantly decreased in the M group. Notably, Esc treatment significantly upregulated the expression of these proteins, whereas this protective effect was markedly attenuated by pretreatment with CC. These results suggest that the protective effect of Esc on AML12 cells is mediated, at least in part, through the activation of the AMPK/SIRT1/PGC-1α signaling pathway.

## 4. Discussion

The liver is a major metabolic organ that plays essential roles in deoxidation, glycogen storage, and protein synthesis [20]. Due to the lack of effective treatments, uncontrolled acute liver injury often leads to irreversible damage and compensatory repair, resulting in liver fibrosis and eventually cirrhosis or liver cancer [21]. Therefore, the development of effective therapeutic strategies for ALI remains urgently needed [22]. Esc is a coumarin compound widely present in Chinese herbal medicines [23,24] and exerts diverse pharmacological effects, including antioxidation, anti-inflammation, and anti-tumor activities [25,26]. The present study verified the hepatoprotective efficacy of Esc against LPS/D-GalN-induced ALI and systematically explored its underlying molecular mechanisms.

Serum ALT and AST are widely used indicators of liver function [27]. These two enzymes are primarily found within liver cells. When liver cells are damaged, leading to changes in membrane permeability, they are released into the bloodstream in large quantities, causing a significant increase in serum transaminase activity [28]. Therefore, they can serve as sensitive biomarkers for liver injury. Our results showed that Esc significantly reduced serum ALT, AST, and TBA levels and improved histopathological damage in liver tissues. HE staining further demonstrated that Esc alleviated hepatocyte necrosis and preserved liver architecture, indicating its hepatoprotective effects in vivo.

Oxidative stress and subsequent apoptosis are central to the pathogenesis of ALI [29]. However, when liver injury occurs, Kupffer cells and neutrophils produce a large amount of oxygen radicals [30,31]. At this time, the body’s SOD cannot completely eliminate ROS, leading to the production of lipid peroxides such as MDA and the release of a large number of pro-inflammatory cytokines such as TNF-α, IL-1β, and IL-6, further aggravating liver tissue injury [32]. In addition, apoptosis is a key mechanism contributing to hepatocyte loss, mainly mediated by mitochondrial and death receptor pathways [33,34]. In this study, Esc reduced intracellular ROS levels and MitoROS production, stabilized mitochondrial membrane potential, and decreased TUNEL-positive apoptosis in AML12 cells, suggesting its protective effects against oxidative stress and apoptosis.

Mechanistically, the liver often experiences excessive ROS generation, accumulation of reactive metabolites, and mitochondrial dysfunction [35,36]. ROS not only directly induces oxidative stress and hepatocyte death but also activates downstream inflammatory pathways, exacerbating liver injury [37]. The innate immune system also contributes to the progression of ALI [38]. Consistently, Esc significantly reduced ROS levels and TUNEL-positive cells in the M group mice.

As the central metabolic hub, the liver is tightly regulated by multiple energy-sensing signaling pathways. Proteomic sequencing of liver tissues suggested that the AMPK signaling pathway is closely implicated in the pathogenesis of ALI. Studies indicate that AMPK/SIRT1 plays important roles in regulating mitochondrial function and inflammatory responses. AMPK and SIRT1 function as core regulators of cellular energy metabolism and stress adaptation. SIRT1 modulates glucose and lipid metabolism, mitochondrial biogenesis, and inflammatory responses by targeting multiple transcription factors and metabolic enzymes. As a pivotal sensor of cellular energy status, AMPK is activated under low-energy conditions to promote ATP generation and suppress ATP-consuming processes, thereby maintaining energy balance. The SIRT1/AMPK axis participates in glucose and lipid metabolism, oxidative stress response, and autophagy processes by regulating downstream effector molecules such as PGC-1α and FoxO. AMPK enhances SIRT1 activity by increasing intracellular NAD+ levels or activating PGC-1α, which further inhibits the transcription of pro-inflammatory cytokines such as TNF-α and IL-1β, thereby alleviating inflammatory responses [39]. The ROS-mediated inflammatory cascade is a key driver of ALI, and the AMPK/SIRT1 pathway acts as a critical regulatory node in this pathological process [40]. In the present study, Esc markedly upregulated the AMPK/SIRT1/PGC-1α signaling pathway, as verified by Western blot and immunofluorescence. Furthermore, pharmacological inhibition of AMPK partially abolished the hepatoprotective effects of Esc, suggesting that AMPK signaling is at least partially responsible for the protective action of Esc against ALI.

Accumulating evidence indicates that pretreatment with Esculetin effectively attenuates CCl_4_-induced liver injury by normalizing serum LDH and GGT levels and ameliorating hepatic oxidative stress [41]. Furthermore, Esculetin has been shown to mitigate metabolic dysfunction-associated fatty liver disease (MAFLD) by modulating lipotoxicity and lipid accumulation via the JNK and AMPK pathways [15]. Beyond Esculetin, a variety of coumarin derivatives have demonstrated significant hepatoprotective activities. For instance, 4′-O-β-d-glucosyl-5-O-methylvisamminol alleviates acetaminophen-induced acute liver injury by inhibiting ferroptosis and activating the PPAR signaling pathway [28]. Similarly, Wedelolactone, a classic coumestan derivative with potent antioxidant and anti-inflammatory properties, improves cholestatic liver disease through the FXR-bile acid-NF-κB/NRF2 regulatory axis [42]. Collectively, the broad hepatoprotective effects observed across various coumarin compounds further corroborate the potential of Esculetin as a reliable therapeutic agent for acute liver injury. Although this study elucidates the protective role of Esculetin via the AMPK/SIRT1 signaling axis, the pathology of acute liver injury also involves complex processes such as macrophage differentiation [4] and liver regeneration [43], which were not explored in this work. Future studies will focus on investigating the potential synergistic effects between AMPK and these processes. Additionally, the therapeutic efficacy of Esculetin on chronic liver diseases, such as liver fibrosis, remains to be verified. In conclusion, as a natural, safe, and widely distributed phytochemical, Esculetin represents a promising lead compound for the treatment of acute liver injury, and the AMPK/SIRT1 signaling pathway may serve as a novel therapeutic target for liver diseases.

The results demonstrate that Esc effectively alleviates oxidative stress and apoptosis in ALI, and these beneficial effects are associated with the regulation of the AMPK/SIRT1/PGC-1α signaling pathway. This study provides novel experimental evidence and mechanistic insights for exploring the hepatoprotective effect and clinical potential of Esc against ALI.

## Figures and Tables

**Table 1:** The docking score of Esc.

Ligand	Receptor	Binding Energy (kcal/mol)
Esc	AMPK	−6.5
	PGC-1α	−6.4
	SIRT1	−6.2

## Data Availability

The original contributions presented in this study are included in the article. Further inquiries can be directed to the corresponding authors.

## Abbreviations

The following abbreviations are used in this manuscript:

ALI	Acute liver injury
AML12	Alpha mouse liver 12
Esc	Esculetin
LPS	Lipopolysaccharide
D-GalN	D-(+)-galactosamine hydrochloride
HE	Hematoxylin and eosin
WB	Western blot
JC-1	JC-1 mitochondrial membrane potential dye
ROS	Reactive oxygen species
TUNEL	Terminal deoxynucleotidyl transferase dUTP nick-end labeling
ALT	Alanine transaminase
AST	Aspartate transaminase
TBA	Total bile acid
AMPK	Adenosine monophosphate-activated protein kinase
FBS	Fetal bovine serum
P/S	Penicillin–streptomycin
ITS-G	Insulin–transferrin–selenium-G
GO	Gene Ontology
KEGG	Kyoto Encyclopedia of Genes and Genomes
BSA	Bovine serum albumin
